# Posterior Arthroscopic Treatment of a Massive Effusion in the Flexor Hallucis Longus Tendon Sheath Associated with Stenosing Tenosynovitis and Os Trigonum

**DOI:** 10.1155/2020/6236302

**Published:** 2020-03-27

**Authors:** Ichiro Tonogai, Koichi Sairyo

**Affiliations:** Department of Orthopedics, Institute of Biomedical Science, Tokushima University Graduate School, 3-18-15 Kuramoto, Tokushima, Tokushima 770-8503, Japan

## Abstract

We report a rare case of massive accumulation of fluid in the flexor hallucis longus tendon sheath with stenosing tenosynovitis and os trigonum. A 34-year-old woman presented to our hospital with pain and swelling in the posteromedial aspect of the left ankle joint after an ankle sprain approximately 8 months earlier. There was tenderness at the posteromedial aspect of the ankle, and the pain worsened on dorsiflexion of the left great toe. Magnetic resonance imaging revealed massive accumulation of fluid around the flexor hallucis longus tendon. We removed the os trigonum, performed tenosynovectomy around the flexor hallucis longus, and released the flexor hallucis longus tendon via posterior arthroscopy using standard posterolateral and posteromedial portals. At 1 week postoperatively, the patient was asymptomatic and able to resume her daily activities. There has been no recurrence of the massive accumulation of fluid around the flexor hallucis longus tendon as of 1 year after the surgery. To our knowledge, this is a rare case report of extreme massive effusion in the flexor hallucis longus tendon sheath with stenosing tenosynovitis and os trigonum treated successfully by removal of the os trigonum, tenosynovectomy around the flexor hallucis longus, and release of the flexor hallucis longus tendon via posterior ankle arthroscopy.

## 1. Introduction

The flexor hallucis longus (FHL) tendon passes through a tendon sheath extending from the flexor retinaculum at the posterior talus through a fibroosseous tunnel along the medial calcaneus to the inferior aspect of the sustentaculum tali. The thick FHL tendon sheath lies just near the medial aspect of the talar process. If the os trigonum is present, it is usually entrapped and narrowed under the thick tendon sheath. Stenosing tenosynovitis of the FHL with involvement of the os trigonum has been reported as a major pathologic finding [1–4].

Effusion in the FHL tendon is often associated with stenosing tenosynovitis. Magnetic resonance imaging (MRI) is useful for assessing the degree of effusion and can confirm the diagnosis by the presence of excess fluid around the FHL in the region of the fibroosseous tunnel [5–7]. However, massive effusion around the FHL tendon is rare.

Here, we report on a patient who presented with massive effusion in the FHL tendon sheath with stenosing tenosynovitis involving an os trigonum, which was successfully treated by resection of the os trigonum, tenosynovectomy around the FHL, and release of the FHL tendon via posterior ankle arthroscopy. To our knowledge, this is a rare case report of extreme massive effusion around the FHL.

## 2. Case Report

**Figure 1:**
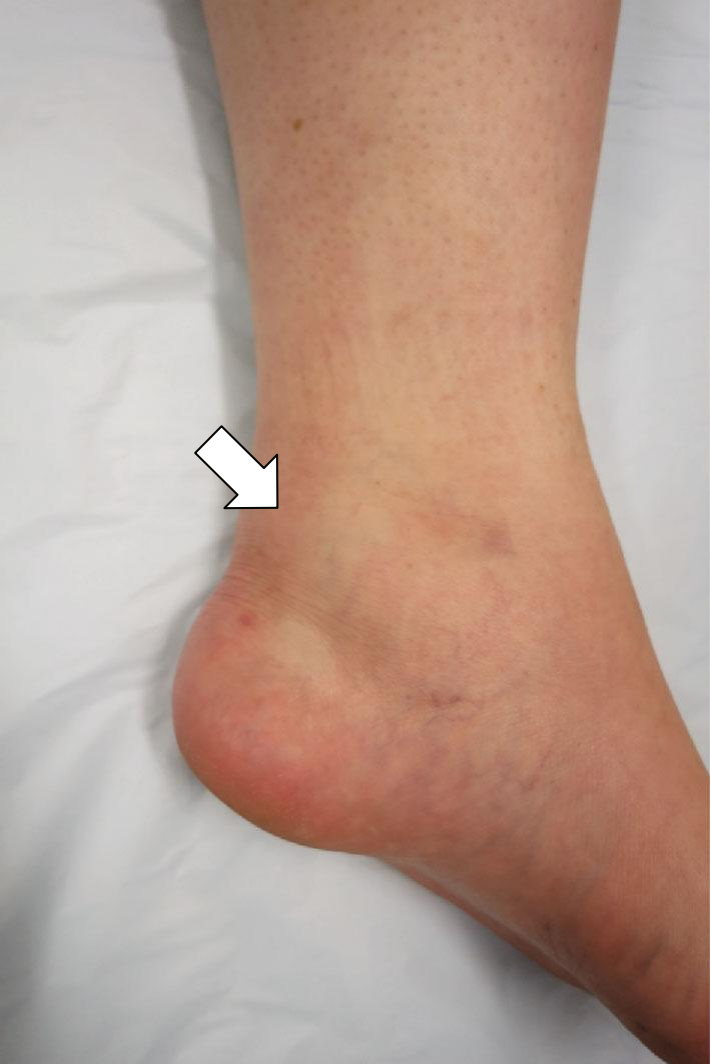
Preoperative photograph showing swelling of the posteromedial side of the left ankle (white arrow).

**Figure 2:**
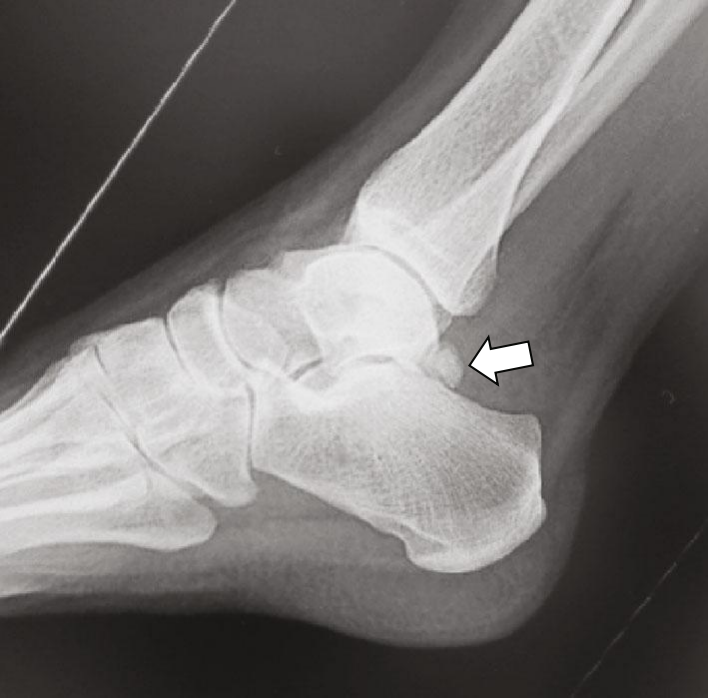
Lateral radiograph showing the os trigonum (arrow).

**Figure 3:**
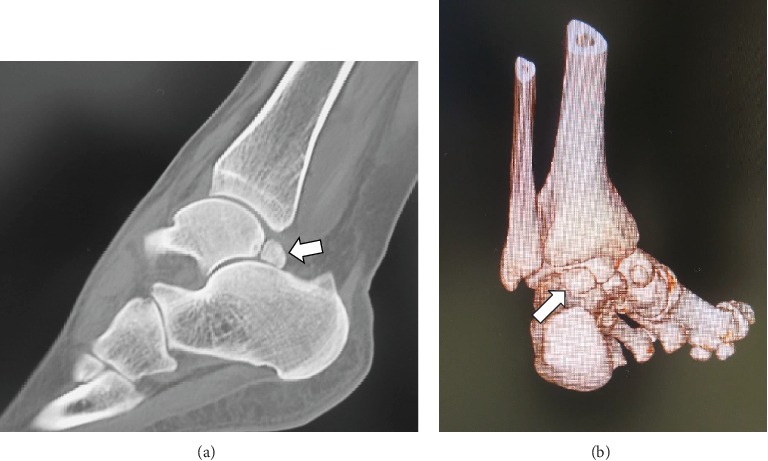
Computed tomography images showing the os trigonum connected to the posterior aspect of the talus. (a) Plain image, sagittal view (arrow). (b) Three-dimensional image, posterior view (arrow).

**Figure 4:**
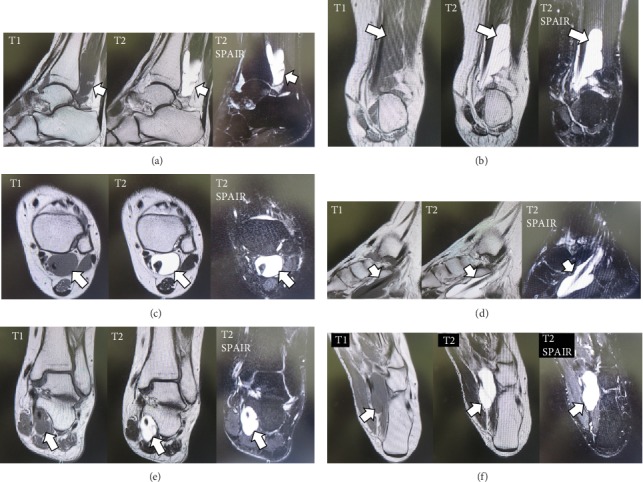
T1-weighted, T2-weighted, and T2-spectral attenuated inversion recovery magnetic resonance images showing a massive effusion around the flexor hallucis longus (arrow) at a level proximal to the ankle joint posteriorly in the (a) sagittal, (b) coronal, and (c) axial planes and between the talar tubercles and the master knot of Henry in the (d) sagittal, (e) coronal, and (f) axial planes.

A 34-year-old woman was referred to our department with an 8-month history of pain and swelling of the left ankle after sustaining an ankle sprain while descending a flight of stairs. She had no significant past medical history. Physical examination revealed swelling and tenderness on the medial side of the left ankle (Figure 1). There was slight limitation of range of motion at the ankle. The ankle pain worsened on dorsiflexion of the left great toe. No neurovascular deficit was noted. Her JSSF (Japanese Society for Surgery of the Foot) scale score was 69/100 (pain 20/40, function 39/50, and alignment 10/10). The patient rated her pain as 6/10 on a visual analogue scale (VAS). An os trigonum was visible on a weight-bearing lateral radiograph of the left ankle (Figure 2) and on a computed tomography (CT) scan (Figures 3(a) and 3(b)). MRI revealed massive effusion around the FHL tendon at a level proximal to the ankle joint posteriorly (Figures 4(a)–4(c)) and between the talar tubercles and the master knot of Henry (Figures 4(d)–4(f)). The preoperative diagnosis was massive effusion around the FHL with stenosing tenosynovitis and os trigonum. Initially, we injected a steroid and xylocaine into the posterior ankle space under ultrasound guidance. The pain decreased but returned within a few days and became persistent. Therefore, we proceeded to a surgical arthroscopic procedure.

**Figure 5:**
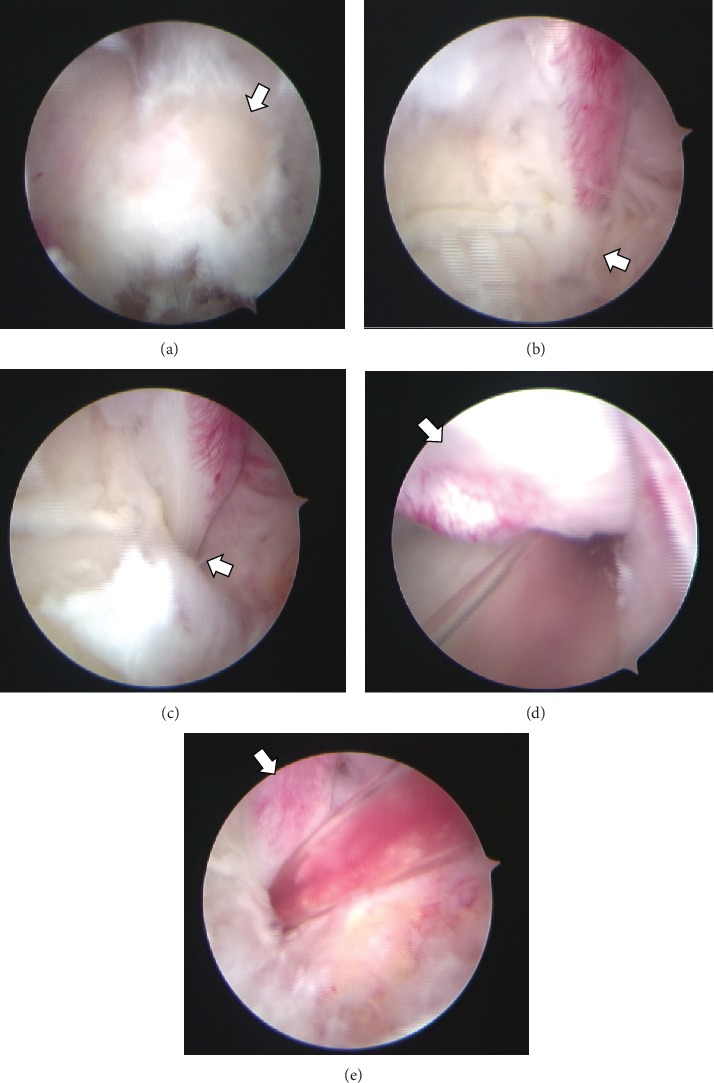
Arthroscopic views of the posterior aspect of the left ankle. (a) The posterior aspect of the os trigonum (arrow) can be visualized. (b) The fibrous portion of the tunnel compressed the flexor hallucis longus (FHL) tendon (arrow) after removal of the os trigonum. (c) Stenosis was not seen (arrow) after the release of the FHL tendon. Suction was performed along the FHL tendon (d) toward a level proximal to the ankle joint posteriorly and (e) anterior to the talar tubercles and the master knot of Henry.

The patient was positioned prone with a thigh tourniquet. Two portals were made 1 cm above the insertion of the Achilles tendon just medial and lateral to the tendon in line with the tip of the lateral malleolus, and then, we performed a posterior hindfoot endoscopy using the standard 2-portal technique described by van Dijk et al. [1]. The lateral portal was used mainly for visualization, and the medial one served as the working portal. A 4 mm 30-degree arthroscope was introduced through the portals and directed toward the second toe. The posterior aspect of the talus and os trigonum was then visualized (Figure 5(a)). The os trigonum was impinged between the posterior aspect of the talus and the calcaneus. The os trigonum on the posteromedial aspect of the talus was resected with a motorized shaver to visualize the entire FHL tendon sheath. The FHL was thick, and a fibroosseous tunnel was seen adherent to the tendon (Figure 5(b)). All fibrous tissues compressing the FHL were cut and removed with a motorized shaver. Next, the tendon was released (Figure 5(c)). Suction was performed along the FHL tendon toward a level proximal to the ankle joint posteriorly (Figure 5(d)) and anterior to the talar tubercles and the master knot of Henry (Figure 5(e)). There were no intraoperative complications.

**Figure 6:**
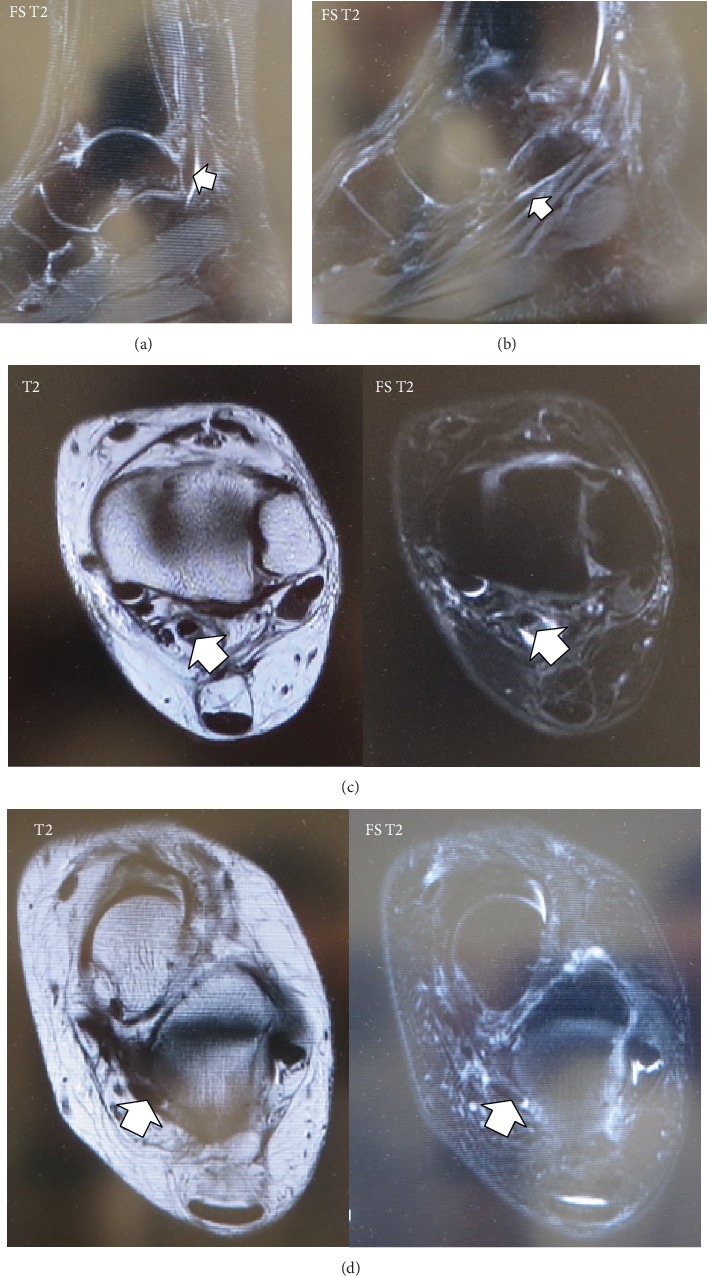
Fat saturation T2-weighted magnetic resonance images showing no effusion along the flexor hallucis longus (FHL) tendon at a level proximal to the ankle joint posteriorly in the (a) sagittal and (b) axial planes. T2-weighted and fat saturation T2-weighted images showing no accumulation of effusion along the FHL between the talar tubercles and the master knot of Henry in the (c) sagittal and (d) axial planes.

After skin closure, a bulky dressing was placed without immobilization. The patient was encouraged to actively move the ankle and toes. Weight bearing was allowed after surgery as tolerated with return to normal daily activities after a week. The postoperative course was unremarkable. At the 1-year follow-up visit, the patient remained asymptomatic and MRI showed no recurrence of the fluid around the FHL (Figures 6(a)–6(d)). Her JSSF scale score had improved from 69/100 points to 100/100 points and her VAS score to 0/10.

The patient provided informed consent for the publication of this report.

## 3. Discussion

We have reported the case of a 34-year-old woman who presented with a massive effusion along the FHL tendon sheath with stenosing tenosynovitis and os trigonum. Although FHL tenosynovitis often occurs in the os trigonum syndrome, an extreme effusion along the FHL tendon is extremely rare.

The FHL is a secondary producer of torque at the ankle subtalar joint complex and hallux joints [8–10] but functions primarily as an active plantar flexor at the first metatarsophalangeal and hallux interphalangeal joints [11–13] and provides primary restraint to passive dorsiflexion at the first metatarsophalangeal joint [6, 7, 10, 14]. Given the finding by Hamilton and Chao that there is relative incongruity between the FHL and the fibroosseous tunnel when the foot is in extreme plantar flexion [7], it is possible that the FHL tendon was subjected to abnormal stresses in our patient when she sustained a severe left ankle sprain. Alternatively, she might have an anatomic feature whereby the FHL tendon enters the tunnel at an oblique angle, predisposing it to irritation. As documented by Sammarco and Cooper [6], FHL tendinitis is not uncommon, even in nonathletes, and should always be considered in the differential diagnosis of posteromedial ankle pain.

The os trigonum syndrome with stenosing FHL tenosynovitis is a common cause of posterior ankle impingement, as encountered in our patient. Conservative treatment is the recommended first line of treatment. Secondary treatment options are open or arthroscopic excision of the os trigonum with release of the FHL tendon. Open surgery for the os trigonum associated with tenosynovitis of the FHL has been reported [15]. However, arthroscopic approaches have gained popularity in the past decade because they produce less scarring and are associated with less postoperative pain, minimal overall morbidity, and an earlier return to daily activity. A more rapid return to activity, improved visualization of pathology, less postoperative pain, and a decreased risk of complications have been noted as advantages of the endoscopic approach to the posterior hindfoot and ankle [16]. Moreover, some authors have reported the effectiveness of posterior ankle arthroscopy for FHL stenosing tenosynovitis with the os trigonum [17, 18]. Therefore, we selected posterior ankle arthroscopy in this case and found it to be an effective procedure for the treatment of massive effusion caused by stenosing tenosynovitis of the FHL with the os trigonum.

There are several articles of similar reports of massive effusion in the FHL tendon sheath. Qu et al. reported cases of diffuse FHL tenosynovitis with effusion at a level proximal to the ankle joint posteriorly and/or between the talar tubercles and the master knot of Henry, although it was treated by open extensive tenosynovectomy [19]. Lee also reported cases of effusion with the FHL tendon sheath at a level of the interphalangeal joint and/or the metatarsophalangeal joint, although it was treated by an open procedure [20]. However, we treated a massive effusion in the FHL tendon sheath with stenosing tenosynovitis involving an os trigonum via posterior ankle arthroscopy. This point might be different from reports by Qu et al. [19] and Lee et al. [20].

One limitation of this report is the short follow-up period. Although the effusion has not recurred as of the latest follow-up visit 1 year after surgery, further follow-up is necessary.

In conclusion, we encountered a rare case of massive effusion along the FHL tendon associated with stenosing tenosynovitis and os trigonum that was treated by removal of the os trigonum and release of the stenosing fibrous tunnel via a posterior arthroscopic procedure. Surgical treatment was successful in this case even with massive effusion along the FHL tendon sheath.
